# Effects of Intake of Fish or Fish Oils on the Development of Diabetes

**DOI:** 10.14740/jocmr1964w

**Published:** 2014-10-16

**Authors:** Hidekatsu Yanai, Hidetaka Hamasaki, Hisayuki Katsuyama, Hiroki Adachi, Sumie Moriyama, Akahito Sako

**Affiliations:** a Department of Internal Medicine, National Center for Global Health and Medicine Kohnodai Hospital, Chiba, Japan

**Keywords:** Diabetes, Docosahexaenoic acid, Eicosapentaenoic acid, Fish, Insulin sensitivity

## Abstract

The association between fish and fish oils intake and diabetes remains largely unknown. Here we systematically reviewed published articles (clinical trials, prospective cohort studies, systematic reviews and meta-analyses) about the effects of intake of fish or fish oils on the development of diabetes. An intake of fish oils seems not to affect insulin sensitivity, insulin secretion, beta-cell function or glucose tolerance. There is a considerable statistical heterogeneity in the overall summary estimates of the association between fish or fish oils consumption and the development of type 2 diabetes, which is partly explained by geographical differences. Marine n-3 polyunsaturated fatty acids have beneficial effects on the prevention of type 2 diabetes in Asian populations.

## Introduction

Epidemiological and clinical studies indicate a significant inverse association between intake of fish oils, eicosapentaenoic acid (EPA) and docosahexaenoic acid (DHA), and mortality associated with coronary artery disease [1-6]. Secondary prevention trials reported that increased consumption of fish or fish-oil supplements reduced coronary death in post-infarction patients [7, 8]. The proposed anti-atherosclerotic properties of EPA include reduction of platelet aggregation [9], amelioration of endothelial dysfunction [10], suppression of the proliferation of vascular smooth muscle cells [11], stabilization of fatty plaque [12] and reduction of fasting blood triglyceride [13].

Evidences to show beneficial effects of fish oils on atherosclerotic diseases have been accumulating; however, the association between fish or fish oils intake and diabetes remains largely unknown. Here we systematically reviewed published articles about the effects of intake of fish or fish oils on the development of diabetes.

## Clinical Trials and Prospective Cohort Studies to Show Effects of Intake of Fish or Fish Oils on the Development of Diabetes

Clinical trials and prospective cohort studies about effects of intake of fish or fish oils on the development of diabetes were shown in Table 1 [14-20]. Giacco et al performed the clinical trial to evaluate the effect of a moderate supplementation of long-chain n-3 fatty acids (FA) on insulin sensitivity, insulin secretion, beta-cell function and glucose tolerance in healthy individuals [14]. One hundred sixty-two healthy individuals were randomly assigned to diets rich in monounsaturated fats and the other rich in saturated fats for 3 months. Within each group there was a second randomization to fish oil (n-3 FA 3.6 g/day) or placebo. A moderate supplementation of fish oil did not affect insulin sensitivity, insulin secretion, beta-cell function or glucose tolerance. Griffin et al studied the effect of dietary ratio of n-6 to n-3 (n-6:n-3) polyunsaturated fatty acids (PUFA) on metabolic parameters [15]. In a randomized, parallel design in 258 subjects aged 45 - 70 years old, four diets providing 6% of energy as PUFA with an n-6:n-3 between 5:1 and 3:1 with a control diet that had an n-6:n-3 of 10:1 were compared. Changes in the n-6:n-3 did not influence insulin sensitivity.

**Table 1:** Clinical Trials and Prospective Cohort Studies to Show Effects of Intake of Fish or Fish Oils on the Development of Diabetes

Authors	Study design	Subjects studied	Results/conclusions
Giacco et al [14]	Healthy individuals were randomly assigned to monounsaturated fats and the other rich in saturated fats for 3 months. Within each group there was a second randomization to fish oil (n-3 fatty acids 3.6 g/day) or placebo.	162 healthy individuals. Performed in Sweden.	A moderate supplementation of fish oil does not affect insulin sensitivity, insulin secretion, beta-cell function or glucose tolerance.
Griffin et al [15]	In a randomized, parallel design, four diets providing 6% of energy as PUFA with an n-6:n-3 between 5:1 and 3:1 with a control diet that had an n-6:n-3 of 10:1 for 6 months were compared. The diets were enriched in ALA, EPA and DHA, or both.	258 subjects aged 45 - 70 years old. Performed in UK.	Decreasing the n-6:n-3 does not influence insulin sensitivity.
Brostow et al [16]	To examine the association between total omega-3 FA, marine omega-3 (EPA, DHA), nonmarine omega-3 (ALA), and omega-6 FA and omega-6:omega-3 ratio and risk of type 2 diabetes.	43,176 Chinese men and women free of chronic disease, aged 45 - 74 years old, in the Singapore Chinese Health Study.	Omega-3 FA from marine sources were not associated with diabetes risk.
Villegas et al [17]	A prospective population-based cohort study. A Cox regression model was used to evaluate the association of fish, shellfish, and long-chain n-3 FA with risk of type 2 diabetes.	51,963 Chinese men and 64,193 Chinese women free of type 2 diabetes, cardiovascular diseases, and cancer.	An inverse association between fish and shellfish intake and type 2 diabetes in women was found.
Djousse et al [18]	A prospective study. Incident type 2 diabetes was self-reported and validated primarily through the collection of supplementary information from participants. Information on omega-3 and fish intakes was obtained by using a validated food-frequency questionnaire. Cox proportional hazard models were used to estimate adjusted RR.	36,328 women (mean age: 54.6 years old) who participated in the Women’s Health Study and who were followed from 1992 to 2008. Performed in USA.	An increased risk of type 2 diabetes with the intake of long-chain omega-3 FA, especially with higher intakes (≥ 0.20 g omega-3/day or ≥ 2 servings of fish/day).
Van Woudenbergh et al [19]	A population-based cohort. Hazard ratios (RR) with 95% CIs were used to examine risk associations adjusted for age, sex, lifestyle, and nutritional factors.	4,472 Dutch participants aged ≥ 55 years old without diabetes.	A beneficial effect of total fish, type of fish, or EPA and DHA intake on the risk of type 2 diabetes was not observed.
Djousse et al [20]	Plasma phospholipid n-3 FA were measured by using gas chromatography, and incident diabetes was ascertained by using information on hypoglycemic agents and serum glucose. Cox proportional hazards models were used to estimate multivariable-adjusted relative risks.	3,088 older men and women (mean age: 75 years old) from the Cardiovascular Health Study (1992 - 2007). Performed in USA.	Long-chain n-3 FA were not associated with a higher incidence of diabetes.

ALA: α-linolenic acid; CI: confidence interval; DHA: docosahexaenoic acid; EPA: eicosapentaenoic acid; FA: fatty acids; PUFA: polyunsaturated fatty acids; RR: relative risk.

Brostow et al examined the association between total omega-3 FA, EPA, DHA, α-linolenic acid, and omega-6 FA and omega-6:omega-3 ratio and risk of type 2 diabetes in a Chinese population in Singapore [16]. Intake of omega-3 FA from marine sources (EPA and DHA) was not associated with diabetes risk. Villegas et al performed a prospective population-based cohort study in 51,963 men and 64,193 women free of type 2 diabetes, cardiovascular diseases, and cancer, to examine the associations between fish, shellfish, and long-chain n-3 FA and the risk of type 2 diabetes in a middle-aged Chinese population [17]. Fish, shellfish, and long-chain n-3 FA intakes were inversely associated with type 2 diabetes in women. The relative risks (RRs (95% CI)) for quintiles of fish intake were 1.00, 0.96 (0.86 - 1.06), 0.84 (0.75 - 0.94), 0.80 (0.71 - 0.90), and 0.89 (0.78 - 1.01) (P for trend = 0.003) and for shellfish were 1.00, 0.91 (0.82 - 1.01), 0.79 (0.71 -0.89), 0.80 (0.71 - 0.91), and 0.86 (0.76 - 0.99) (P = 0.006). In men, only the association between shellfish intake and type 2 diabetes was significant. The RRs (95% CI) for quintiles of fish intake were 1.00, 0.92 (0.75 - 1.13), 0.80 (0.65 - 1.00), 0.89 (0.72 - 1.11), and 0.94 (0.74 - 1.17) (P for trend = 0.50) and for shellfish intake were 1.00, 0.93 (0.76 - 1.12), 0.70 (0.56 - 086), 0.66 (0.53 - 0.82), and 0.82 (0.65 - 1.02) (P for trend = 0.003).

Djousse performed a prospective study of 36,328 women (mean age: 54.6 years old) who participated in the Women’s Health Study and who were followed from 1992 to 2008, to evaluate effects of dietary omega-3 FA and fish consumption on the risk of type 2 diabetes [18]. From the lowest to highest quintiles of marine omega-3 intake, the multivariable-adjusted hazard ratios (95% CIs) for type 2 diabetes were 1.0 (referent), 1.17 (1.03 - 1.33), 1.20 (1.05 - 1.38), 1.46 (1.28 - 1.66), and 1.44 (1.25 - 1.65), respectively (P for trend < 0.0001), suggesting an increased risk of type 2 diabetes with the intake of long-chain omega-3 FA, especially with higher intakes (≥ 0.20 g omega-3 FA/day or ≥ 2 servings of fish/day). Van Woudenbergh et al investigated the relation between total fish, type of fish (lean and fatty), and EPA and DHA intake and risk of type 2 diabetes in a population-based cohort study, by the analysis including 4,472 Dutch participants aged ≥ 55 years old without diabetes [19]. Total fish intake was positively associated with risk of type 2 diabetes; the RR was 1.32 (95% CI 1.02 - 1.70) in the highest total fish group (≥ 28 g/day) compared with that for non-fish eaters (P for trend = 0.04). Lean fish intake tended to be associated positively with the development of type 2 diabetes (RR highest group (≥ 23 g/day) 1.30 (95% CI 1.01 - 1.68), P for trend = 0.06), but fatty fish was not. No association was observed between EPA and DHA intake and type 2 diabetes. Djousse et al examined the relation between plasma phospholipid n-3 FA and incident diabetes, by the analysis of 3,088 older men and women (mean age: 75 years old) from the Cardiovascular Health Study (1992 - 2007) [20]. Long-chain n-3 FA was not associated with a higher incidence of diabetes.

## Meta-Analyses Investigating the Effects of Intake of Fish or Fish Oils on the Development of Diabetes

Meta-analyses about effects of intake of fish or fish oils on the development of diabetes were shown in Table 2 [21-24]. Akinkuolie et al systematically reviewed the effect of n-3 PUFA on insulin sensitivity by conducting a meta-analysis of available randomized controlled trials (RCTs) [21]. Eleven RCTs (n = 618) were eligible for inclusion in the analysis. In a pooled estimate, n-3 PUFA intervention had no effects on insulin sensitivity compared to placebo.

**Table 2:** Meta-Analyses to Show Effects of Intake of Fish or Fish Oils on the Development of Diabetes

Authors	Study design	Subjects studied	Results/Conclusions
Akinkuolie et al [21]	To systematically review the effect of n-3 PUFA on insulin sensitivity by conducting a meta-analysis of available RCTs. MEDLINE, EMBASE, CENTRAL and clinicaltrials.gov from the beginning of each database until October 2010 were used.	Eleven RCTs (n = 618) were eligible for inclusion in the analysis.	This meta-analysis is consistent with a lack of n-3 PUFA effects on insulin sensitivity.
Wallin et al [22]	To find the evidence on the association between fish consumption, dietary long-chain n-3 FA, and risk of type 2 diabetes, studies were identified by searching the PubMed and EMBASE databases through December 15, 2011 and by reviewing the reference lists of retrieved articles.	Sixteen studies involving 527,441 participants and 24,082 diabetes cases were included.	For each serving per week increment in fish consumption, the RRs (95% CIs) of type 2 diabetes were 1.05 (1.02 - 1.09), 1.03 (0.96 - 1.11), and 0.98 (0.97 - 1.00) combining US, European, and Asian/Australian studies, respectively. For each 0.30 g per day increment in long-chain n-3 FA, the corresponding summary estimates were 1.17 (1.09 - 1.26), 0.98 (0.70 - 1.37), and 0.90 (0.82 - 0.98), respectively.
Zheng et al [23]	A systematic review and meta-analysis of prospective cohort studies to examine the associations of fish and n-3 PUFA intake with type 2 diabetes risk.	PubMed, Embase, Cochrane library, China National Knowledge Infrastructure (CNKI) and Chinese VIP database up to January 2012 were used to identify relevant studies.	Marine n-3 PUFA have beneficial effects on the prevention of type 2 diabetes in Asian populations.
Wu et al [24]	Systematically search for multiple literature databases through June 2011 to identify prospective studies examining relations of dietary n-3 PUFA, dietary fish and/or seafood, and circulating n-3 PUFA biomarkers with incidence of diabetes.	Sixteen studies including 18 separate cohorts comprising 540,184 individuals and 25,670 cases of incident diabetes.	The overall pooled findings do not support either major harms or benefits of fish/seafood or EPA + DHA on development of diabetes.

CI: confidence interval; DHA: docosahexaenoic acid; EPA: eicosapentaenoic acid; FA: fatty acids; PUFA: polyunsaturated fatty acids; RCT: randomized controlled trial.

Wallin et al performed a systematic review and meta-analysis to understand the association between fish consumption, dietary n-3 FA, and risk of type 2 diabetes, by searching the PubMed and EMBASE databases through December 15, 2011 [22]. Sixteen studies involving 527,441 participants and 24,082 diabetes cases were included. Considerable statistical heterogeneity in the overall summary estimates was partly explained by geographical differences. For each serving per week increment in fish consumption, the RRs (95% CIs) of type 2 diabetes were 1.05 (1.02 - 1.09), 1.03 (0.96 - 1.11), and 0.98 (0.97 - 1.00), in combined analysis of studies performed in US, European, and Asian/Australian, respectively. For each 0.30 g per day increment in long-chain n-3 FA, the corresponding summary estimates were 1.17 (1.09 - 1.26), 0.98 (0.70 - 1.37), and 0.90 (0.82 - 0.98). This meta-analysis indicated differences between geographical regions in observed associations of fish consumption and dietary intake of long-chain n-3 FA with risk of type 2 diabetes. Zheng et al conducted a systematic review and meta-analysis of prospective cohort studies to examine the associations of fish and n-3 PUFA intake with type 2 diabetes risk [23]. Twenty-four studies including 24,509 type 2 diabetic patients and 545,275 participants were identified. For cohort studies, the summary RR of type 2 diabetes for the highest vs. lowest categories of total fish and marine n-3 PUFA intake was 1.07 (95% CI: 0.91 - 1.25) and 1.07 (95% CI: 0.95 - 1.20), respectively. Subgroup analyses indicated that summary RR (highest vs. lowest category) of type 2 diabetes for fish and marine n-3 PUFA intake was 0.89 (95% CI: 0.81 - 0.98) and 0.87 (95% CI: 0.79 - 0.96) for Asian populations, and 1.20 (95% CI: 1.01 - 1.44) and 1.16 (95% CI: 1.04 - 1.28) for Western populations. This systematic review and meta-analysis showed that marine n-3 PUFA have beneficial effects on the prevention of type 2 diabetes in Asian populations. Wu et al systematically searched multiple literature databases through June 2011 to identify prospective studies examining relations of dietary n-3 PUFA, dietary fish and/or seafood, and circulating n-3 PUFA biomarkers with incidence of diabetes [24]. Sixteen studies met inclusion criteria, including 18 separate cohorts comprising 540,184 individuals and 25,670 cases of incident diabetes. Consumption of fish and/or seafood was not significantly associated with diabetes (n = 13 studies; RR per 100 g/day = 1.12, 95% CI = 0.94 - 1.34), nor was consumption of EPA + DHA (n = 16 cohorts; RR per 250 mg/day = 1.04, 95% CI = 0.97 - 1.10) nor circulating levels of EPA + DHA biomarkers (n = 5 cohorts; RR per 3% of total fatty acids = 0.94, 95% CI = 0.75 - 1.17). In unadjusted meta-regressions, study location (Asia vs. North America/Europe), mean BMI, and duration of follow-up each modified the association between fish/seafood and EPA + DHA consumption and DM risk (P-interaction ≤ 0.02 each).

## Conclusion

An intake of fish oils seems not to affect insulin sensitivity, insulin secretion, beta-cell function or glucose tolerance. There is a considerable statistical heterogeneity in the overall summary estimates of the association between fish consumption, dietary long-chain n-3 FA, and risk of type 2 diabetes, which is partly explained by geographical differences. Marine n-3 PUFA have beneficial effects on the prevention of type 2 diabetes in Asian populations.
